# Complete mitochondrial genomic sequence of *Lenzites betulinus* (Polyporales, Basidiomycota)

**DOI:** 10.1080/23802359.2026.2722781

**Published:** 2026-09-03

**Authors:** Yunping Wang, Guoxiang Zhong, Qiping Zhong, Jiping Ma, Yinrun Xiao, Suzhen Wang, Xiaowen Xiong, Cheng Zhang

**Affiliations:** Institute of Agricultural Applied Microbiology, Jiangxi Academy of Agricultural Sciences, Nanchang, China

**Keywords:** Mitochondrial genome, Polyporaceae, phylogeny, mushrooms

## Abstract

*Lenzites betulinus* is a medicinal fungus in the Polyporaceae. Here, we report the first complete mitochondrial sequencing of this species. The circular genome is 61,288 bp in length, with a GC content of 26.32% and base composition of A (36.98%), T (36.70%), G (12.96%), and C (13.36%). It contains 14 core protein-coding genes (PCGs), 26 tRNA genes, two rRNA genes, and nine intronic ORFs within the cox1 gene. A maximum-likelihood phylogenetic tree based on 14 PCGs from 17 mitochondrial genomes confirmed that *L. betulinus* clusters within the Polyporaceae, closely related to *Trametes* and *Fomitopsis*. This served as a significant reference for ongoing research into other species within the Polyporales order.

## 1. Introduction

**Figure 1.**
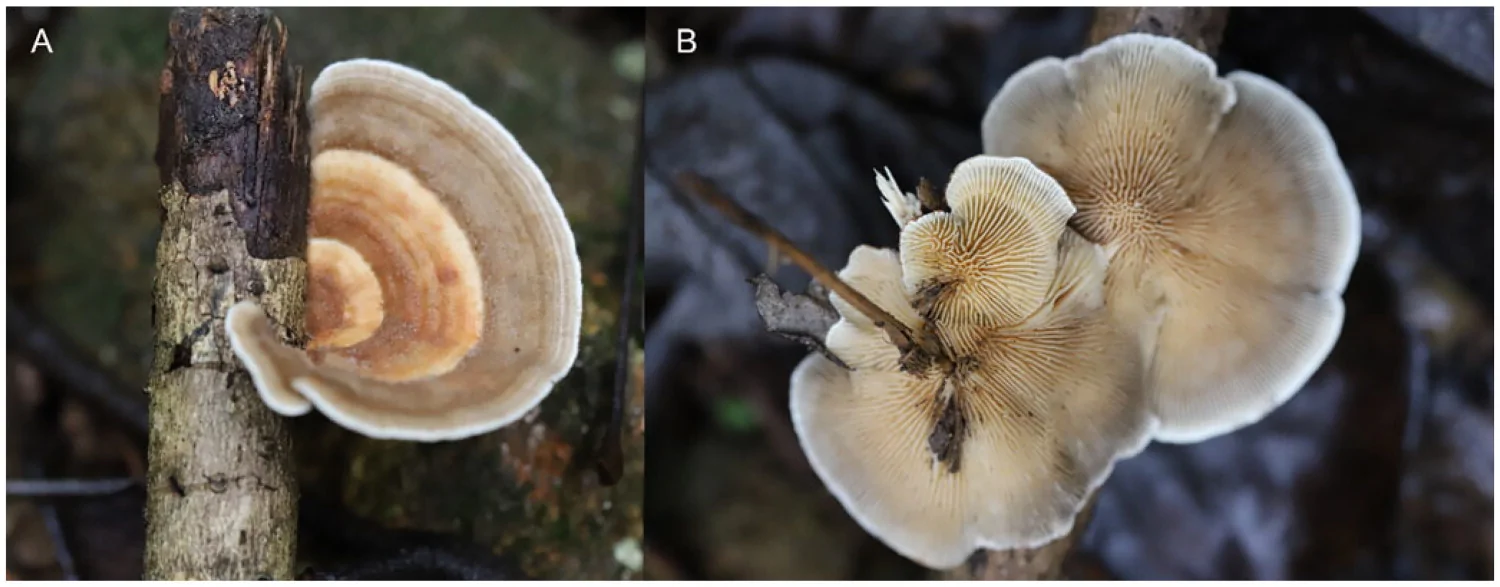
Photos of the *L. betulinus* specimen used in this study. (A) A topside view of the *L. betulinus* fruiting body; (B) an underside view of the *L. betulinus* fruiting body. Photographed by Yinrun Xiao (one of the authors) at the Institute of Agricultural Applied Microbiology; Jiangxi Academy of Agricultural Sciences, Nanchang, China.

*Lenzites betulinus* (L.) Fr. 1838 is a species of bracket fungus within the order Polyporales and family Polyporaceae that typically produces small, pileate-sessile fruiting bodies annually (Cui et al. 2019; Zhao et al. 2024). The pileus is commonly dimidiated, semicircular, or flabelliform in shape. It is characterized by concentric sulcations and striations, particularly along the margin. This margin may be even, lobed, or frequently exhibiting a wavy outline (Li et al. 2015) (see Figure 1). *L. betulinus* is found and thrives on diverse types of deciduous wood and is widely distributed across Asia and Australia (Li et al. 2015). Historically, this fungus has been used in traditional medicine to alleviate conditions including haunch and femoral pain, acropathy, apoplexy, and colds (Ren et al. 2006). Recent research suggests that *L. betulinus* could offer potential protection against therapy-induced DNA damage in peripheral blood lymphocytes of patients suffering from acute coronary syndrome (Vukajlovi et al. 2024). Due to its remarkable health benefits, extensive research has been conducted on the extraction, functional identification, and clinical applications of its bioactive compounds (Fakoya and Oloketuyi 2012).

Mitochondrial genomes have been widely used as molecular markers in phylogenetic studies. Compared to nuclear genomes, they generally have a low recombination rate, conserved gene content, a maternal inheritance mechanism, and mitochondrial DNA is relatively easy to isolate and sequence (Rubinoff and Holland 2005; Smith 2016). Thus, many studies have utilized mtDNA to determine the phylogenetic and evolutionary relationships among fungal species (Chen et al. 2019; Li et al. 2019; Fonseca et al. 2020). Despite the increasing research on the mitochondrial genome of Polyporaceae, the complete mitochondrial genome of *L. betulinus* and the genus *Lenzite*s remains unexplored. Current studies on *L. betulinus* primarily emphasize its chemical composition and pharmacological properties (Fakoya and Oloketuyi 2012). This gap in information significantly limits our understanding of the phylogenetic relationships and the evolutionary history within *Lenzites*.

Consequently, this study determined the mitochondrial genome information of *L. betulinus*, analyzing the sequence characteristics, gene composition, and phylogenetic relationships of this species using bioinformatics software. This study establishes a theoretical foundation for investigating the genetic structure and diversity of *L. betulinus*, while also facilitating future phylogenetic analyses and research on genetic diversity in the Polyporaceae family.

## 2. Materials and methods

### 2.1. Materials

Samples of *L. betulinus* were collected from Fuzhou, Jiangxi Province, China (27.29° N, 116.90° E). Voucher specimens (No. 20231027004) were deposited at the Institute of Applied Agricultural Micro-Organisms (contact Jiping Ma, 455735885@qq.com), Jiangxi Academy of Agricultural Sciences. The species was identified through a combination of morphological investigation and internal transcribed spacer (*ITS*) sequencing (Figure 1).

### 2.2. DNA sequencing, genome assembly, and annotation

Genomic DNA samples were isolated from the fruiting bodies with a DNA Kit (OMEGA, Norcross, GA). The complete mitochondrial genome was sequenced using the NovaSeq 6000 platform (BIOZERON Co., Ltd, Shanghai, China), employing a paired-end 150 bp sequencing strategy. Before assembly, raw reads were filtered by Trimmomatic 0.39 (Bolger et al. 2014). The mitochondrial genome was reconstructed using a combination of *de novo* and reference-guided assembly approaches. The filtered reads were assembled into contigs using GetOrganelle v1.6.4 (Jian et al. 2018), and potential mitochondrial contigs were extracted by aligning against the NCBI mitogenome database (ftp://ftp.ncbi.nlm.nih.gov/refseq/release/mitochondrion/). Mitochondrial genes were annotated using the online MFannot tool (Lang et al. 2023), employing default parameters to identify protein-coding, tRNA, and rRNA genes. Manual corrections of genes for start/stop codons and intron/exon boundaries were performed in SnapGene Viewer by referencing related mitochondrial (NC_054271) genomes. The mt genome map of *L. betulinus* was drawn using the OGDRAW tool (Greiner et al. 2019).

### 2.3. Phylogenetic analysis

Based on the results of BLAST, we downloaded 16 complete Agaricomycetes mitochondrial genomes from the National Center for Biotechnology Information (NCBI) nonredundant (nr) database. A total of 14 homologous protein-coding genes (PCGs) from the 17 fungal taxa underwent multiple alignment using MAFFT v7.037 (Katoh et al. 2019). The phylogenetic estimation model (GTR+F + R2) was selected using ModelTest. Phylogenetic relationships were estimated using maximum-likelihood (ML) approaches as implemented in IQ-TREE v1.6.12 (Nguyen et al. 2015).

## 3. Results

**Figure 2.**
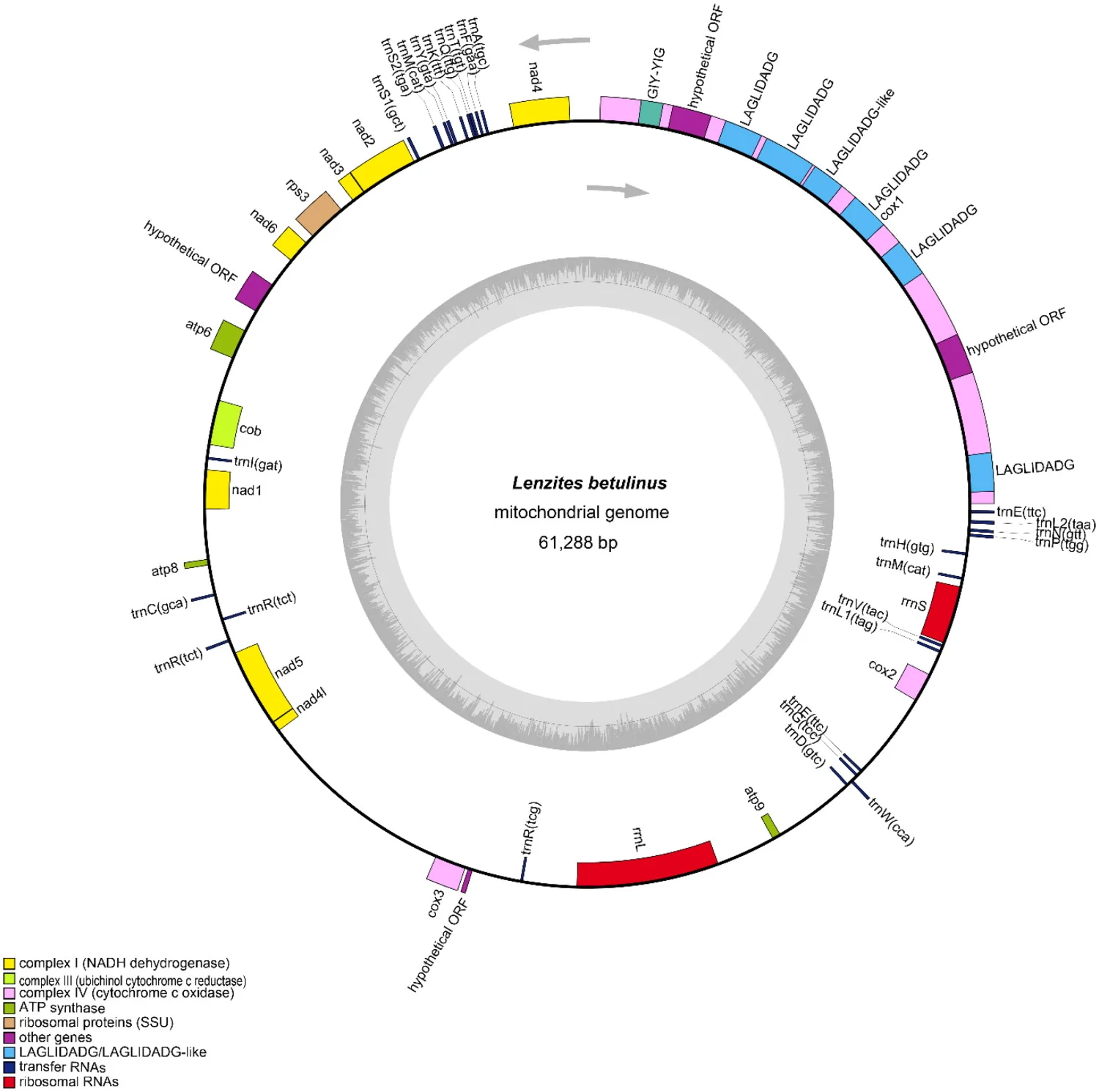
Circular map of the *L. betulinus* mitochondrial genome. Different colored blocks represent various categories of genes. Blocks outside the ring represent forward strand genes and blocks inside the ring represent reverse strand genes. GC and AT contents across the genome are shown with dark and light shading, respectively, inside the inner circle.

**Figure 3.**
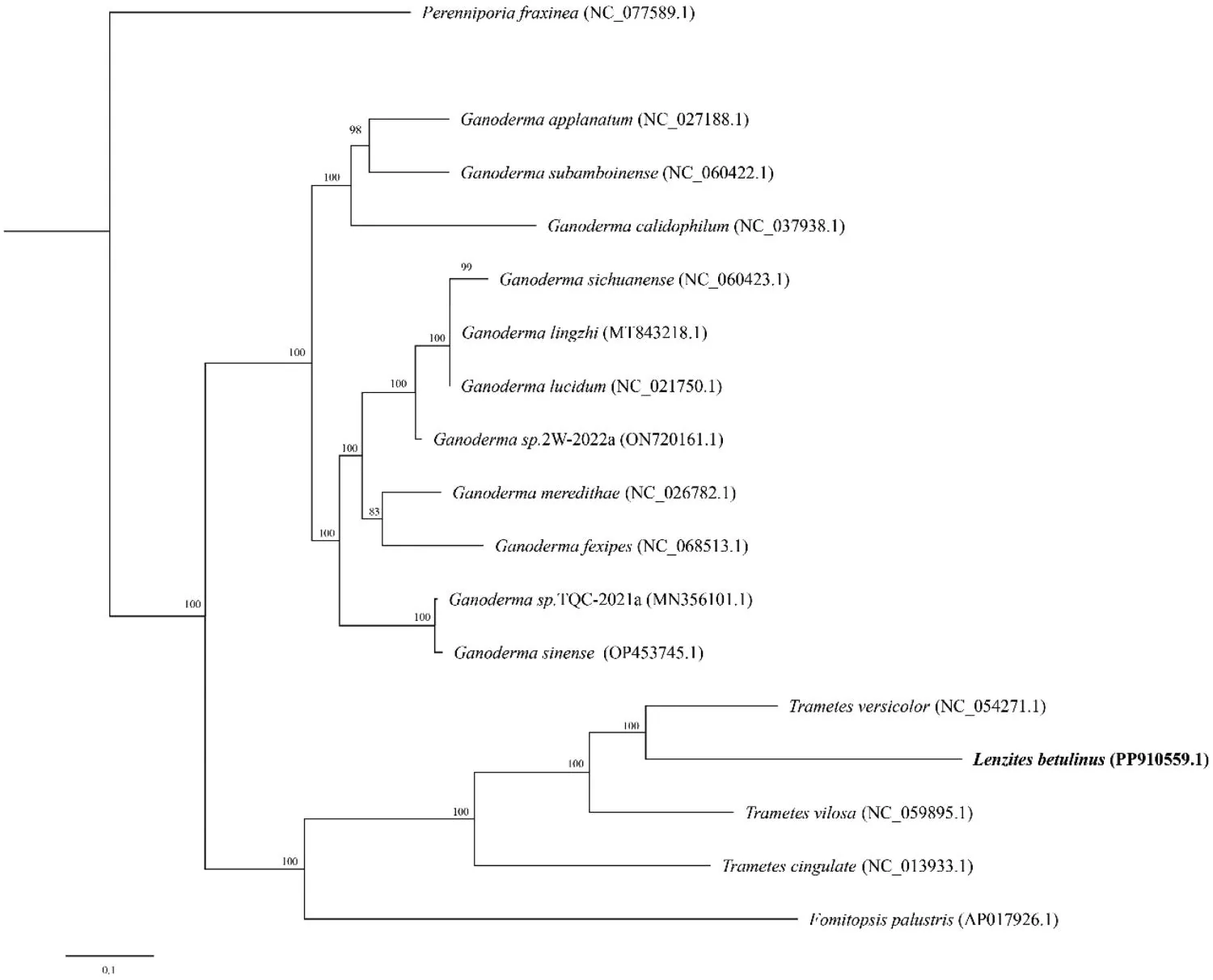
Phylogenetic tree showing 17 species of Agaricomycetes conducted by maximum-likelihood method based on concatenated amino acid sequences of 14 core PCGs, with *Perenniporia fraxinea* as outgroup. Node values indicate bootstrap support (1000 replicates). The complete mitochondrial sequences and accession ID were used as follows: *Perenniporia fraxinea* NC_077589.1 (Cho et al. 2023); *Ganoderma applanatum* NC_027188.1 (Wang et al. 2016); *Ganoderma subamboinense* NC_060422.1 (Li et al. 2022); *Ganoderma calidophilum* NC_037938.1 (Li et al. 2018); *Ganoderma sichuanense* NC_060423.1 (Cho et al. 2023); *Ganoderma lingzhi* MT843218.1 (Meng et al. 2022); *Ganoderma lucidum* NC_021750.1 (Cho et al. 2023); *Ganoderma sp.2W-2022a* ON720161.1 (Cho et al. 2023); *Ganoderma meredithae* NC_026782.1 (Cho et al. 2023); *Ganoderma flexipes* NC_068513.1 (Cho et al. 2023); *Ganoderma sp.* TQC-2021a MN356101.1 (Chen et al. 2024); *Ganoderma sinense* OP453745.1 (Cho et al. 2023); *Trametes versicolor* NC_054272.1 (Chen et al. 2021); *Trametes villosa* NC_059895.1 (Araújo et al. 2021); *Trametes cingulate* NC_013933.1 (Cho et al. 2023); *Fomitopsis palustris* AP017926.1 (Cho et al. 2023).

The average sequencing depth was 3022.17×, as shown in Figure S1. The complete mitochondrial genome sequence of *L. betulinus* (GenBank accession number PP910559) is a circular molecule measuring 61,288 bp in length, with a GC content of 26.32%, and a base composition of A (36.98%), T (36.70%), G (12.96%), and C (13.36%). Based on their sequence lengths, the 14 core PCGs collectively account for 89.46% of the total genome length (61,288 bp), while the 26 tRNA genes and two rRNA genes account for 3.16% and 7.37%, respectively. The *L. betulinus* mitochondrial genome contained 14 core PCGs (89.46%), 26 tRNA genes (3.16%), and two rRNA genes (*rnl* and *rns*) (7.37%). The 14 core PCGs included seven NADH:ubiquinone reductase subunits (*nad1*, *nad2*, *nad3*, *nad4*, *nad4L*, *nad5*, and *nad6*), three subunits of cytochrome C oxidase (*cox1*, *cox2*, and *cox3*), three ATP synthase subunits (*atp6*, *atp8*, and *atp9*), and apocytochrome b (*cob*) (Figure 2). Additionally, the *cox1* gene contains eight introns, which harbor nine intronic ORFs, including six LAGLIDADG/LAGLIDADG-like homing endonuclease genes, one GIY-YIG homing endonuclease gene, and two uncharacterized hypothetical ORFs (as confirmed by NCBI CD-Search). Two additional stand-alone hypothetical ORFs, which contain no conserved domains, were identified outside the cox1 introns. Based on a phylogenetic analysis using 14 core PCGs, the ML phylogenetic tree revealed that several fungal species, including *Trametes versicolor* (NC_054272.1), *L. betulinus*, *Trametes villosa* (NC_059895.1), *Trametes cingulate* (NC_013933.1), and *Fomitopsis palustris* (AP017926.1), form a distinct clade. Within this clade, *L. betulinus* clusters with *Trametes versicolor* (NC_054271.1) (Figure 3), confirming its close phylogenetic relationship with the genera *Trametes* and *Fomitopsis*.

## 4. Discussion and conclusions

GetOrganelle (https://github.com/Kinggerm/GetOrganelle) is a software tool designed for organelle genome assembly, particularly well suited for assembling fungal mitochondrial genomes (Jian et al. 2020). After assembly using GetOrganelle, it was further refined and corrected to yield the complete mitochondrial genome sequence of *L. betulinus*. Our sequencing results showed a high level of sequencing depth. The complete mitogenomes of *L. betulinus* and *T. versicolor* are circular, with sizes of 61,288 bp and 67,318 bp, respectively. Moreover, the *cox1* gene of *L. betulinus* contains eight introns (harboring nine intronic ORFs), while that of *T. versicolor* possesses 14 introns (containing 13 intronic ORFs) (Chen et al. 2021). Phylogenetic analysis indicates that *L. betulinus* and *T. versicolor* are within the same clade, although notable differences in genome size, intron content, and GC content suggest rapid divergence between the two species.

Introns are considered primary contributors to the size expansion of Polyporales mitogenomes, and their dynamic changes are a main factor in mitogenome size variation in this fungal order (Férandon et al. 2010; Himmelstrand et al. 2014; Liu et al. 2020). Comparative analysis revealed that the genome size of *L. betulinus* (61,288 bp, GC 26.32%) is smaller than that of *T. versicolor* (67,318 bp), *T. coccinea* (99,976 bp), and *T. cingulata* (91,500 bp), while the core gene content (14 PCGs, 26 tRNAs, and two rRNAs) remains conserved across these species. The *T. versicolor* mitogenome exhibits more introns than *L. betulinus*, which is consistent with the results of previous studies. Notably, the intronic ORF content also differs between these species: *L. betulinus* harbors nine intronic ORFs within its *cox1* gene (including six LAGLIDADG/LAGLIDADG-like, one GIY-YIG, and two hypothetical ORFs), while T. versicolor contains 13 intronic ORFs (Chen et al. 2021). However, mitogenome length and intron number are not always positively correlated. The complete mitogenomes of *L. betulinus* and *F. palustris*, for example, contain 8 and 6 introns and measure 61,288 bp and 63,479 bp, respectively (Li et al 2018). Although *F. palustris* has fewer introns (six) compared to *L. betulinus* (eight), its mitogenome is longer (63,479 bp). Moreover, mitogenomes within the Polyporales order vary in size and content, even within the same clade. Gene rearrangements, including inversions and insertions, have also been documented among *Trametes* species (Chen et al. 2021), suggesting that both intron dynamics and genomic structural changes shape mitogenome evolution. This supports prior studies indicating that the varied classes and number of introns in Polyporales suggest intron gain/loss occurred during the evolution of the mitogenome (Li et al. 2018; Wang et al. 2020; Megarioti & Kouvelis, 2020).

Mitochondrial genes are widely used as molecular markers for studying phylogenetic relationships among eukaryotes because they offer several advantages over nuclear DNA (Boore 1999; Cameron 2014). However, the availability of complete mitochondrial genomes in public databases like NCBI (https://www.ncbi.nlm.nih.gov/genome/browse#!/overview/) and JGI (http://genome.jgi.doe.gov/programs/fungi/index.jsf) remains relatively limited, especially for certain taxa such as fungi. Consequently, our study represents the first complete mitochondrial genome of *L. betulinus*, contributing to the existing repository of mitochondrial genomes within the Polyporales. Furthermore, the results from the phylogenetic analysis offer valuable insights that will facilitate a more comprehensive understanding of Polyporales, which will be further clarified as more mitochondrial genomes are sequenced.

## Supplementary Material

Supplemental Material

## Data Availability

The mitochondrial genome sequence data that support the findings of this study are openly available in GenBank of NCBI at https://www.ncbi.nlm.nih.gov/ under accession no. PP910559. The associated BioProject, SRA, and Bio-Sample numbers are PRJNA1140544, SRR30004227, and SAMN42835698, respectively.
