# Supplementary material for: Complete mitochondrial genomic sequence of Lenzites betulinus (Polyporales, Basidiomycota)
Source: Mitochondrial DNA B Resour. 2026 Sep 3;11(10):1100–5. doi: 10.1080/23802359.2026.2722781 (PMC13543834; doi:10.1080/23802359.2026.2722781)
Supplement: Supplementary file 1 [file TMDN_A_2722781_SM7596.docx]

**Supplemental material**

Complete mitochondrial genome sequence of *Lenzites betulinus* (Polyporales, Basidiomycota)

Supplementary material:

Figure S1. Sequencing depth map for the mitochondrial genome of *Lenzites betulinus* (The X-axis indicates the location of bases in the genome; the Y-axis indicates the coverage depth).
